# Eating Behavior Patterns, Food Group Intake Frequency, and Sarcopenia in Community‐Dwelling Older Adults: A Cross‐Sectional Study From the Kashiwa Study

**DOI:** 10.1002/hsr2.72832

**Published:** 2026-07-16

**Authors:** Tomoki Tanaka, Fumika Kamijo, Hiroko Ito, Koji Kurata, Katsuya Iijima

**Affiliations:** ^1^ Institute of Gerontology The University of Tokyo Tokyo Japan; ^2^ Institute for Future Initiatives The University of Tokyo Tokyo Japan; ^3^ Kewpie Corporation Tokyo Japan

**Keywords:** eating behavior patterns, food group intake frequency, nutritional care, older adults, sarcopenia

## Abstract

**Background and Aim:**

A protein‐rich and diverse diet is essential for maintaining muscle health, but issues such as eating alone, shopping difficulties, and reduced appetite negatively impact dietary habits in older adults. This study aimed to examine the relationship between eating behavior patterns, food group intake frequency, dietary variety and sarcopenia among community‐dwelling older adults.

**Methods:**

The study included 2004 men and women (72.9 ± 5.5 year old) from the Kashiwa Study, excluding those with cognitive impairment or missing data. Eating behaviors were classified into four clusters using latent class analysis based on 13 eating‐related indicators. Dietary content was evaluated by the frequency of intake across 10 food groups and the dietary variety score (DVS). Sarcopenia was diagnosed using AWGS 2019, and logistic regression analysis compared its prevalence among the clusters.

**Results:**

The four clusters were: (1) No preparation difficulty, normal appetite type (64.8%, no difficulties, normal food intake), (2) No preparation difficulty, high appetite type (20.0%, large meal size, enjoys meals), (3) No preparation difficulty, low appetite type (11.4%, low appetite), and (4) Preparation‐difficulty type (3.8%, difficulty in meal preparation). The DVS did not differ significantly across the four clusters. However, specific food group intake frequencies differed by cluster; the Preparation‐difficulty type had lower intake frequencies of meat, seafood, soybean products, and fruits. Sarcopenia risk was lower in the No preparation difficulty, high appetite type (odds ratio 0.7 [0.4–1.3]) but significantly higher in the No preparation difficulty, low appetite type (2.8 [1.9–4.3]) and Preparation‐difficulty type (2.3[1.2–4.3]).

**Conclusions:**

Eating behavior patterns, such as difficulty with meal preparation and low appetite, are related to specific food group intake frequencies and sarcopenia. These findings suggest that multidimensional assessment of eating behavior may help identify older adults who could benefit from nutritional assessment and support.

## Summary

- Distinct eating behavior patterns were associated with food group intake frequency and sarcopenia.
- Low appetite and preparation difficulties were associated with higher odds of sarcopenia.
- Although overall Dietary Variety Score did not differ significantly across clusters, specific food group intake frequencies differed, particularly among those with meal preparation difficulty.

## 1 Introduction

As the global population ages, Japan stands out with 29% of its population aged 65 or older as of 2023, far exceeding the World Health Organization's (WHO) threshold for a “super‐aged society.” Japan's aging rate remains among the highest in developed countries. As this population grows, maintaining independence and extending the healthy lifespans of older adults is critical. Sarcopenia, the age‐related decline in muscle mass and strength, increases the risk of falls, fractures, and frailty [1]. Therefore, preventing sarcopenia is essential for promoting healthy aging and preventive care. Adequate energy and protein intake, along with a diverse diet, are key factors in preventing sarcopenia and frailty [2, 3].

In addition to diet, various eating behavior challenges—such as eating alone, food access difficulties, and reduced appetite—affect food choices and dietary diversity in older adults [4, 5, 6]. Given the wide individual differences in eating habits and nutrient intake, personalized attention is necessary. Although many studies have explored the relationship between dietary issues and sarcopenia or frailty [7, 8, 9], most focus on a single dietary factor. In reality, dietary challenges are multifaceted, but few studies provide a comprehensive view of eating behaviors in older adults. Latent class analysis may be useful for identifying naturally occurring patterns of multidimensional eating‐related behaviors rather than evaluating each factor separately.

This study aims to address this gap by classifying the eating behavior patterns of community‐dwelling older adults through latent class analysis. Additionally, it investigates how these patterns are associated with dietary content, sarcopenia, and related factors, including skeletal muscle mass, muscle strength, and physical performance. These findings may help characterize eating behavior profiles associated with sarcopenia and inform future approaches to nutritional assessment and support in aging populations.

## 2 Methods

### 2.1 Study Setting and Participants

This cross‐sectional study used baseline data from the 2012 Kashiwa Study, a prospective cohort study of older adults in Kashiwa, Chiba [10] (References). Of the approximately 80,000 people aged 65 and older who had not been certified as needing long‐term care, 12,000 were randomly selected, and 2044 (with a 1:1 male‐to‐female ratio) participated in the baseline survey. Participants with complete data on eating behaviors and sarcopenia, and without suspected cognitive impairment (Mini‐Mental State Examination, MMSE ≥ 18), were included in the analysis.

This study adhered to the Strengthening the Reporting of Observational Studies in Epidemiology (STROBE) guidelines for observational studies. The study protocol was approved by the ethics committee of the University of Tokyo (#21‐192), and written informed consent was obtained from all participants. This study was conducted in accordance with the principles of the Declaration of Helsinki.

### 2.2 Measures

#### 2.2.1 Eating Behavior and Awareness

Eating behavior was evaluated using responses to the following 13 items: daily food intake (a lot/somewhat a lot/normal/somewhat little/little), snack amount (same categories), number of meals per day, eating speed (fast/normal/slow), drinking habits (yes/quit/no), supplement use (yes/no), difficulties in shopping (yes/no), difficulties in meal preparation (yes/no), ability to prepare meals alone (yes/no), daily meal companionship (yes/no), dining with friends at least once a month (yes/no), appetite (yes/no), and enjoyment of meals (yes/no).

#### 2.2.2 Diversity of Food Intake

Participants completed a self‐administered questionnaire to assess their weekly intake of 10 food groups: seafood, meat, eggs, dairy, soybean products, green and yellow vegetables, seaweed, fruits, tubers and root crops, and oils and fats. Responses were scored on a 4‐point scale (almost every day/once every 2 days/1–2× per week/almost never). A diversity variety score (DVS) was calculated by assigning 1 point for each food group consumed “almost every day,” with a maximum possible score of 10 points [3]. Because the DVS is based on food group intake frequency, it does not directly measure total energy intake, protein intake, or other nutrient intakes.

#### 2.2.3 Physical Functions and Sarcopenia

Sarcopenia was diagnosed using the criteria set by the Asian Working Group for Sarcopenia 2019 (AWGS 2019) [11]. Skeletal Muscle Mass Index (SMI) was measured using bioelectrical impedance analysis (BIA) with an InBody 420 body composition analyzer (InBody Japan, Tokyo, Japan). SMI was calculated by dividing limb muscle mass by height squared, with low muscle mass defined as < 7.0 kg/m^2^ for men and < 5.7 kg/m^2^ for women. Muscle strength was measured with a Grip D dynamometer (Takei Scientific Instruments, Niigata, Japan), and the maximum value from two measurements was used. Low muscle strength was defined as < 28 kg for men and < 18 kg for women. Walking speed was measured over 5 meters, with a speed of < 1 m/s considered slow [11].

#### 2.2.4 Basic Attributes

Participants' basic attributes included age, gender, Body Mass Index (BMI), living arrangements, social isolation (LSNS‐6) [12], depression (Geriatric Depression Scale‐15, GDS‐15) [13], cognitive function (MMSE) [14], oral health‐related quality of life (General Oral Health Assessment Index, GOHAI) [15], and the number of remaining teeth. Nurses also interviewed participants about their history of chronic diseases, including hypertension, diabetes, osteoporosis, hyperlipidemia, cancer, heart disease, stroke, and chronic renal failure).

### 2.3 Statistical Analysis

Statistical analyses were conducted using JMP 16 (SAS Institute Inc., Cary, NC, USA), with significance set at *p* < 0.05. All statistical tests were two‐sided. Latent class analysis was used to categorize eating behavior patterns based on 13 items, Models with two to five classes were estimated and compared using Bayesian information criterion (BIC), Akaike information criterion (AIC), class proportions, and interpretability. Although the three‐class solution showed the lowest BIC and the five‐class solution showed the lowest AIC, the four‐class model was selected based on the overall balance of model fit, class size, and clinical interpretability. In particular, the four‐class model enabled identification of clinically meaningful eating behavior profiles, including a distinct preparation‐difficulty type, without excessive fragmentation of classes. Model fit indices for candidate latent class models are shown in Supporting Information S1: Table S1. Participants were assigned to the class with the highest probability. Latent classes were compared with basic attributes and eating behaviors using the chi‐square test for categorical variables and the Kruskal‐Wallis test for continuous variables. Significant differences were further analyzed using residual analysis or Steel‐Dwass test. Logistic regression was used to examine the relationship between latent class and sarcopenia, adjusting for gender, age, education, living arrangements, exercise habits, MMSE scores, and medical history. Confounding‐adjusted odds ratios with 95% confidence intervals were calculated. Additionally, an analysis of covariance (ANCOVA) was performed to examine physical functions—grip strength, walking speed, and SMI—as outcome variables. Gender‐specific analyses were conducted using age, education, living arrangements, exercise habits, MMSE scores, and medical history as covariates.

## 3 Results

### 3.1 Study Participants

Among the 2044 participants who completed the baseline assessment, 40 were excluded due to missing data, resulting in 2004 eligible individuals (mean age: 72.9 ± 5.5 years; 992/2004 [49.5%] men) for analysis. The basic characteristics of the participants are summarized in Table 1.

**Table 1:** Basic characteristics of participants.

		Total	Male	Female
		(*n* = 2004)	(*n* = 992)	(*n* = 1012)
Basic attributes				
Gender	Male (%)	49.5	—	—
Age	Years	72.9 ± 5.5	73.1 ± 5.6	72.8 ± 5.4
BMI	kg/m^2^	22.9 ± 3.0	23.3 ± 2.8	22.5 ± 3.2
Living arrangement	Alone (%)	10.9	5.7	16.0
Years of education	Years	12.7 ± 2.8	13.6 ± 2.9	11.8 ± 2.3
Exercise habits	Yes (%)	77.3	80.3	74.3
Physical and mental condition				
LSNS‐6 score		16.2 ± 5.9	15.9 ± 5.8	16.4 ± 5.9
GDS‐15 score		2.6 ± 2.9	2.4 ± 3.0	2.8 ± 2.9
MMSE score		28.2 ± 1.8	28.2 ± 1.8	28.2 ± 1.8
Remaining teeth		20.6 ± 8.6	20.6 ± 8.9	20.6 ± 8.3
GOHAI score		54.6 ± 6.4	54.6 ± 6.2	54.5 ± 6.7
Medical history				
Hypertension	Yes (%)	43.9	47.4	40.4
Stroke	Yes (%)	6.0	7.2	4.8
Diabetes	Yes (%)	12.1	15.5	8.7
Hyperlipidemia	Yes (%)	38.3	29.7	46.6
Osteoporosis	Yes (%)	11.2	2.0	20.1
Chronic renal failure	Yes (%)	0.7	0.9	0.6
Heart disease	Yes (%)	17.5	21.0	14.0
Cancer	Yes (%)	15.0	19.0	11.2

*Note:* Values are presented as mean ± standard deviation for continuous variables and *n* (%) for categorical variables.

Abbreviations: BMI, Body Mass Index, GDS‐15, Geriatric Depression Scale‐15; GOHAI, General Oral Health Assessment Index; LSNS‐6, Lubben Social Network Scale‐6; MMSE, Mini‐Mental State Examination.

### 3.2 Classification of Eating Behavior Patterns Using Latent Class Analysis

Latent class analysis of the responses to 13 items related to eating behaviors is summarized in Supporting Information S1: Tables S1 and S2. The number of classes was determined by comprehensively examining the BIC and AIC values, composition ratios, and cluster interpretation, resulting in four distinct classes. Models with two to five classes were compared (Supporting Information S1: Table S1). Although the three‐class solution showed the lowest BIC and the five‐class solution showed the lowest AIC, the four‐class solution was selected based on the overall balance of model fit, class size, and clinical interpretability. Conditional response probabilities for the selected four‐class model are shown in Supporting Information S1: Table S2. Cluster 1, the largest group, had a high percentage of participants who reported normal food intake and eating speed, with no difficulties in meal preparation. This group was termed the “No preparation difficulty, normal appetite type.” Cluster 2 had a high proportion of individuals who consumed large meals, ate snacks frequently, enjoyed eating, and had a strong appetite. This cluster was also characterized by a high probability of fast eating speed. This group was labeled the “No preparation difficulty, high appetite type.” Cluster 3 included those who ate small portions, ate slowly, had reduced appetite, and did not enjoy eating, earning the designation “No preparation difficulty, low appetite type.” This cluster also showed higher probabilities of eating alone and eating with friends less than once a month. Cluster 4, characterized by difficulties in meal preparation despite normal food intake, was called the “Preparation‐difficulty type.” This cluster was particularly distinguished by shopping difficulties and meal preparation difficulties, while appetite and daily food intake were relatively preserved.

Although appetite and meal preparation difficulty were major distinguishing characteristics, the latent classes were derived from the joint distribution of all 13 eating‐related indicators. Therefore, the identified classes represented multidimensional eating behavior profiles rather than simple categorization based on a single factor.

Table 2 presents a comparison of basic attributes across the clusters. The “No preparation difficulty, high appetite type” had a higher BMI compared to the other groups, while the “No preparation difficulty, low appetite type” had a lower BMI. Participants in the “No preparation difficulty, low appetite type” and “Preparation‐difficulty type” were older and had higher GDS scores, indicating greater levels of depression. The “No preparation difficulty, normal appetite type” had the lowest proportion of participants living alone, while the “No preparation difficulty, low appetite type” had the highest. Differences were also observed between clusters regarding the history of chronic illness.

**Table 2:** Basic characteristics according to eating behavior patterns.

		No preparation difficulty, normal appetite type	No preparation difficulty, high appetite type	No preparation difficulty, low appetite type	Preparation‐difficulty type	*p*‐value
		(*n* = 1299)	(*n* = 400)	(*n* = 229)	(*n* = 76)	
Gender	Male (%)	49.2	51.0	52.8	36.8	*p* = 0.01
Age	Years	72 [68–76]^a^	71 [68–75]^b^	75 [70–79.5]^c^	76.5 [71–81]^c^	*p* < 0.001
BMI	kg/m2	22.7 [20.9–24.5]^a^	24.2 [22.1–26.0]^b^	21.0 [19.0–23.4]^c^	22.8 [20.6–24.4]^a^	*p* < 0.001
Living arrangements	Alone (%)	9.1	11.8	19.2	13.2	*p* < 0.001
Years of education	Years	12 [12–15]^a^	12 [12–16]^a^	12 [10–14]^ab^	12 [9–13]^b^	*p* < 0.001
Exercise habits	Yes (%)	79.6	77.5	69.87	59.21	*p* < 0.001
LSNS score		17 [12–20]	16 [12–20]	16 [12–20]	15 [12–21]	*p* = 0.82
GDS‐15 score		1 [0–3]^a^	2 [0–4]^a^	3 [1–6]^b^	4 [1.75–8]^b^	*p* < 0.001
MMSE score		29 [27–30]	29 [27–30]	28 [27–29]	28 [26–29]	*p* = 0.02
Remaining teeth	Tooth/teeth	24 [17–27]^a^	25 [20–27]^b^	23 [12–27]^ac^	20 [8–25.5]^c^	*p* < 0.001
GOHAI score		57 [51–60]	57 [52–60]	57 [52–60]	55.5 [50.3–59]	*p* = 0.33
Medical History						
Hypertension	Yes (%)	42.4	46.8	42.4	57.9	*p* = 0.03
Stroke	Yes (%)	4.9	6.3	9.6	11.8	*p* = 0.006
Diabetes	Yes (%)	10.5	13.8	18.4	11.8	*p* = 0.005
Hyperlipidemia	Yes (%)	37.6	42.5	35.4	35.5	*p* = 0.23
Osteoporosis	Yes (%)	10.3	8.3	15.4	29.0	*p* < 0.001
Chronic renal failure	Yes (%)	0.9	0.8	0.4	0.0	*p* = 0.79
Heart disease	Yes (%)	15.9	16.0	27.1	23.7	*p* < 0.001
Cancer	Yes (%)	14.2	14.0	23.6	9.2	*p* = 0.001

*Note:* Categorical variables are expressed as percentages (Chi‐square test). Continuous variables are expressed as median [interquartile range] (Kruskal‐Wallis test). When significant differences were observed for continuous variables, pairwise comparisons were performed using the Steel–Dwass test. a, b, c: Different letters indicate statistically significant differences between groups in post hoc comparisons.

Abbreviations: BMI, Body Mass Index, GDS‐15, Geriatric Depression Scale‐15; GOHAI, General Oral Health Assessment Index; LSNS‐6, Lubben Social Network Scale‐6; MMSE, Mini‐Mental State Examination.

### 3.3 Relationship Between Eating Behavior Patterns, Food Intake Frequency, and Food Intake Diversity

Table 3 presents the comparison of food intake frequency and diversity across clusters. The “No preparation difficulty, normal appetite type” had a high intake frequency of soybeans and fruits. The “No preparation difficulty, high appetite type” showed a high intake frequency of meat, oils, and fats, while the “No preparation difficulty, low appetite type” had a low intake frequency of oils and fats. The “Preparation‐difficulty type” reported low intake frequencies of meat, seafood, soybeans, and fruits. However, when comparing food intake diversity scores, no significant differences were found among the four clusters. Thus, the dietary differences observed across eating behavior patterns were primarily related to specific food group intake frequencies rather than overall DVS.

**Table 3:** Food group intake frequency and dietary variety score according to eating behavior patterns.

Food category	Frequency	Total		No preparation difficulty, normal appetite type		No preparation difficulty, high appetite type		No preparation difficulty, low appetite type		Preparation‐difficulty type		*p*‐value
		*N*	(%)	*N*	(%)	*N*	(%)	*N*	(%)	*N*	(%)	
Meat	Almost every day	334	(16.7)	200	(15.4)*	85	(21.3)*	40	(17.5)	9	(11.8)	*p* = 0.04
	Once every 2 days	590	(29.5)	385	(29.7)	122	(30.5)	66	(28.8)	17	(22.4)	
	Less than twice a week	1079	(53.9)	713	(54.9)	193	(48.3)*	123	(53.7)	50	(65.8)*	
Seafood	Almost every day	457	(22.8)	271	(20.9)*	107	(26.8)*	63	(27.5)	16	(21.1)	*p* = 0.005
	Once every 2 days	793	(39.6)	547	(42.1)*	140	(35.0)*	85	(37.1)	21	(27.6)*	
	Less than twice a week	754	(37.6)	481	(37.0)	153	(38.3)	81	(35.4)	39	(51.3)*	
Soybeans and soy products	Almost every day	1013	(50.6)	687	(52.9)*	177	(44.3)*	119	(52.0)	30	(39.5)*	*p* = 0.02
	Once every 2 days	629	(31.4)	395	(30.4)	142	(35.5)*	63	(27.5)	29	(38.2)	
	Less than twice a week	362	(18.1)	217	(16.7)*	81	(20.3)	47	(20.5)	17	(22.4)	
Eggs	Almost every day	465	(23.2)	281	(21.6)	107	(26.8)	56	(24.5)	21	(27.6)	*p* = 0.28
	Once every 2 days	615	(30.7)	401	(30.9)	114	(28.5)	77	(33.6)	23	(30.3)	
	Less than twice a week	924	(46.1)	617	(47.5)	179	(44.8)	96	(41.9)	32	(42.1)	
Milk and dairy products	Almost every day	1429	(71.3)	948	(73.0)	269	(67.3)	161	(70.3)	51	(67.1)	*p* = 0.25
	Once every 2 days	271	(13.5)	165	(12.7)	64	(16.0)	28	(12.2)	14	(18.4)	
	Less than twice a week	304	(15.2)	186	(14.3)	67	(16.8)	40	(17.5)	11	(14.5)	
Seaweed	Almost every day	409	(20.4)	261	(20.1)	90	(22.5)	48	(21.0)	10	(13.2)	*p* = 0.24
	Once every 2 days	616	(30.8)	396	(30.5)	132	(33.0)	68	(29.7)	20	(26.3)	
	Less than twice a week	978	(48.8)	641	(49.4)	178	(44.5)	113	(49.3)	46	(60.5)	
Green and yellow vegetables	Almost every day	1365	(68.2)	884	(68.1)	284	(71.0)	150	(65.8)	47	(61.8)	*p* = 0.29
	Once every 2 days	427	(21.3)	285	(21.9)	78	(19.5)	48	(21.1)	16	(21.1)	
	Less than twice a week	211	(10.5)	130	(10.0)	38	(9.5)	30	(13.2)	13	(17.1)	
Tubers and root crops	Almost every day	217	(10.8)	138	(10.6)	38	(9.5)	27	(11.8)	14	(18.4)	*p* = 0.39
	Once every 2 days	659	(32.9)	435	(33.5)	130	(32.6)	74	(32.3)	20	(26.3)	
	Less than twice a week	1126	(56.2)	725	(55.9)	231	(57.9)	128	(55.9)	42	(55.3)	
Fruits	Almost every day	1324	(66.1)	880	(67.7)*	243	(60.8)*	157	(68.9)	44	(57.9)	*p* = 0.04
	Once every 2 days	356	(17.8)	228	(17.6)	81	(20.3)	34	(14.9)	13	(17.1)	
	Less than twice a week	323	(16.1)	191	(14.7)*	76	(19.0)	37	(16.2)	19	(25.0)*	
Oils and fats	Almost every day	541	(27.0)	342	(26.3)	123	(30.8)	56	(24.5)	20	(26.3)	*p* = 0.012
	Once every 2 days	570	(28.4)	360	(27.7)	128	(32.0)	55	(24.0)	27	(35.5)	
	Less than twice a week	893	(44.6)	597	(46.0)	149	(37.3)*	118	(51.5)	29	(38.2)	
Dietary Variety Score	Less than 3 pts	920	(46.1)	599	(46.2)	184	(46.1)	100	(44.1)	37	(48.7)	*p* = 0.64
	3–6 pts	901	(45.1)	588	(45.4)	180	(45.1)	99	(43.6)	34	(44.7)	
	7 pts or more	177	(8.9)	109	(8.4)	35	(8.8)	28	(12.3)	5	(6.6)	
	Average ±SE	3.77	0.05	3.77	0.06	3.80	0.11	3.85	0.14	3.45	0.24	*p* = 0.51

*Note:* Categorical variables are expressed as *n* (%) and compared using the chi‐square test.

* Cells marked with an asterisk indicate statistically significant adjusted residuals in residual analysis. Residual analysis < 0.05 (adjusted residual > |1.96 | ).

### 3.4 Relationship Between Eating Behavior Patterns and Sarcopenia

The odds ratios (OR) for sarcopenia in each cluster are shown in Table 4. Using the “No preparation difficulty, normal appetite type” as the reference (OR = 1), the age‐ and gender‐adjusted ORs were: 0.66 [95% CI: 0.37–1.19] for the “No preparation difficulty, high appetite type,” 2.28 [1.48–3.53] for the “No preparation difficulty, low appetite type,” and 2.22 [1.16–4.25] for the “Preparation‐difficulty type.” The same trends were observed in a model adjusted for additional factors (age, gender, exercise habits, cognitive function, educational background, living arrangements, and history of chronic diseases). In this model, the OR for the “No preparation difficulty, high appetite type” remained low at 0.66 [0.36–1.19], while significantly higher ORs were found for the “No preparation difficulty, low appetite type” (2.12 [1.34–3.34]) and “Preparation‐difficulty type” (2.04 [1.05–3.97]).

**Table 4:** Relationship between eating behavior patterns and sarcopenia.

Eating behavior pattern	Sarcopenia, *n*/*N* (%)	Unadjusted model				Model adjusted by age and gender				Multivariate adjusted model*			
		OR	95% confidence interval		*p*	OR	95% confidence interval		*p*	OR	95% confidence interval		*p*
			min	max			min	max			min	max	
No preparation difficulty, normal appetite type	81/1,299 (6.2)	1.00	—	—	—	1.00	—	—	—	1.00	—	—	—
No preparation difficulty, high appetite type	14/400 (3.5)	0.55	0.31	0.97	*p* = 0.040	0.66	0.37	1.19	*p* = 0.17	0.66	0.36	1.19	*p* = 0.17
No preparation difficulty, low appetite type	39/229 (17.0)	3.09	2.05	4.66	*p* < 0.001	2.28	1.48	3.53	*p* < 0.001	2.12	1.34	3.34	*p* = 0.001
Preparation‐difficulty type	15/76 (19.7)	3.70	2.01	6.79	*p* < 0.001	2.22	1.16	4.25	*p* = 0.016	2.04	1.05	3.97	*p* = 0.037

Abbreviation: OR, odds ratio.

* Adjusted for age, gender, exercise, MMSE, years of schooling, living arrangement, and medical history.

Table 5 shows the comparison of muscle mass, grip strength, and walking speed across clusters, using covariance analysis adjusted for basic attributes (age, gender, exercise habits, cognitive function, educational background, living arrangements, and history of chronic diseases). Skeletal muscle mass was significantly higher for both men and women in the “No preparation difficulty, high appetite type” compared to other groups. Walking speed was significantly lower in women from the “Preparation‐difficulty type” compared to the “No preparation difficulty, normal appetite type” and the “No preparation difficulty, high appetite type.” Additionally, grip strength was significantly lower for both men and women in the “No preparation difficulty, low appetite type” compared to the “No preparation difficulty, normal appetite type” and “No preparation difficulty, high appetite type”.

**Table 5:** Adjusted sarcopenia‐related metrics according to eating behavior patterns.

			No preparation difficulty, normal appetite type	No preparation difficulty, high appetite type	No preparation difficulty, low appetite type	Preparation‐difficulty type	*p*‐value
Male	Skeletal muscle mass	(kg/m^2^)	6.99 ± 0.09^a^	7.28 ± 0.10^b^	6.70 ± 0.10^c^	6.90 ± 0.14^ac^	< 0.0001
	Walking speed	(m/sec)	1.42 ± 0.03	1.42 ± 0.04	1.37 ± 0.04	1.32 ± 0.05	0.047
	Grip strength	(kg)	32.7 ± 0.78^a^	33.7 ± 0.81a	30.9 ± 0.85^b^	31.6 ± 1.18^ab^	< 0.0001
Female	Skeletal muscle mass	(kg/m^2^)	5.86 ± 0.07^a^	6.15 ± 0.07b	5.52 ± 0.08^c^	5.86 ± 0.11^a^	< 0.0001
	Walking speed	(m/sec)	1.37 ± 0.03^a^	1.37 ± 0.03a	1.34 ± 0.03^ab^	1.25 ± 0.04^b^	0.0001
	Grip strength	(kg)	22.3 ± 0.39^a^	22.5 ± 0.44a	20.8 ± 0.48^b^	21.1 ± 0.64^ab^	0.004

*Note:* Values are least squares mean ± standard error, adjusted for age, exercise, years of schooling, Mini‐Mental State Examination (MMSE), living arrangement, and medical history. a, b, c: Different letters indicate statistically significant differences between groups in post hoc comparisons of adjusted least squares means.

## 4 Discussion

The dietary challenges and eating behaviors of community‐dwelling older adults are multifaceted, and understanding these patterns may be useful for identifying nutritional and functional vulnerability related to frailty and sarcopenia. This study explored eating behaviors among community‐dwelling older adults, categorizing them into distinct patterns based on food quantity, appetite, and eating environment. The analysis identified four clusters: “No preparation difficulty, normal appetite type,” “No preparation difficulty, high appetite type,” “No preparation difficulty, low appetite type,” and “Preparation‐difficulty type.” Each cluster revealed specific characteristics in terms of basic attributes and dietary content. Notably, the prevalence of sarcopenia was significantly higher in the “No preparation difficulty, low appetite type” and “Preparation‐difficulty type” types. However, the two groups exhibited distinct differences in diet and physical function: the “No preparation difficulty, low appetite type” was associated with small meal portions and reduced muscle strength, while the “Preparation‐difficulty type” was linked to differences in specific food group intake frequency and a decline in walking speed. Importantly, the overall Dietary Variety Score did not differ significantly across clusters, whereas specific food group intake frequencies differed by eating behavior pattern.

The high prevalence of sarcopenia in the “Preparation‐difficulty type” suggests that nutritional imbalances, reflected by lower intake frequencies of specific food groups, may contribute to the risk of sarcopenia. Previous studies have highlighted the role of inadequate intake of nutrients such as protein, omega‐3 fatty acids, and antioxidants (e.g., vitamins and carotenoids) as contributing factors [16, 17, 18, 19]. However, these nutrients were not directly measured in the present study, and therefore their contribution remains speculative. In this study, although the “Preparation‐difficulty type” consumed a normal quantity of food, they had a lower intake frequency of meat, seafood, soybeans, and fruits compared to other clusters. These findings may indicate fewer opportunities to consume protein‐rich and nutrient‐dense foods, rather than directly demonstrating insufficient protein or energy intake. Interestingly, no differences were observed in egg intake frequency, a common protein source, across clusters. A significant correlation between sarcopenia and decreased walking speed was also observed. Prior research has reported that diets with a high “Dietary Inflammatory Index (DII)” are associated with slower walking speeds and may exacerbate age‐related declines in lower limb performance [20]. Moreover, protein intake combined with antioxidant‐rich foods is associated with reduced risk of frailty [21]. These findings from previous studies provide biological plausibility, but the present results should be interpreted cautiously because quantitative nutrient intake was not assessed.

In contrast, the “No preparation difficulty, low appetite type” characterized by low food intake, had a significantly lower intake frequency of oils and fats, which was associated with reduced muscle mass and grip strength (key diagnostic criteria for sarcopenia). The concept of eating without pleasure has recently been discussed as a distinct eating‐related problem, which may be relevant to the low‐appetite pattern characterized by reduced appetite and lower meal enjoyment [22]. Previous studies have also reported that energy intake is associated with muscle mass [23], suggesting that this group may have dietary characteristics associated with lower nutritional adequacy. However, because total energy and protein intake were not directly measured, the nutritional mechanisms underlying this association could not be determined.

Based on these findings, it is important to consider specific food group intake frequencies and eating context, particularly focusing on protein sources for the “preparation‐difficulty” group, while considering the specific characteristics of each cluster. Food insecurity and food access difficulties have also been associated with geriatric syndromes in older adults, supporting the importance of considering food‐related environmental and functional barriers when interpreting meal preparation difficulty [24]. In addition to raising awareness about the importance of diversity in food intake, providing meal delivery services, shopping assistance, and easy‐to‐prepare food products that promote dietary variety may be useful. For the “No preparation difficulty, low appetite type” group, increasing food intake and meals rich in energy may be relevant for nutritional support, although the effectiveness of such approaches for preventing sarcopenia should be evaluated in future intervention studies. Support measures, such as self‐monitoring tools for protein intake and initiatives to boost appetite through communal dining opportunities, are potentially useful approaches.

When comparing the basic attributes across the four clusters in this study, factors such as living arrangements, disease history, and depression were associated with each cluster. Previous studies have reported that social changes and age‐related diseases can significantly impact dietary habits [25]. Anticipating these shifts in eating behaviors due to lifestyle changes associated with aging and providing timely nutritional support before difficulties arise may be useful for identifying older adults at risk of nutritional or functional decline.

In Japan, where the population is rapidly aging, a population‐wide approach is crucial to support the nutritional health of community‐dwelling older adults. Strengthening collaborations with the food industry, healthcare professionals, and community organizations, alongside promoting self‐care among older adults, may be important for addressing diverse eating behavior patterns. This study also highlighted the utility of the DVS as a simple self‐monitoring tool, although no significant differences were observed between clusters [26]. Nevertheless, clusters with a higher risk of sarcopenia exhibited clear biases in the intake of key food groups, suggesting that targeted nutritional education and easy‐to‐implement interventions could improve self‐management. Identifying distinct eating behavior patterns allows healthcare providers and even non‐professionals, such as family members or community volunteers, to deliver tailored nutritional care. Future research and practice should explore how these patterns can inform the development of scalable, personalized interventions to evaluate their potential contribution to sarcopenia prevention and healthy aging.

The present study was conducted among community‐dwelling older adults in an urban municipality in Japan. Therefore, generalizability to rural populations, institutionalized older adults, and non‐Japanese populations should be interpreted with caution. Nevertheless, eating‐related challenges such as appetite loss, food access difficulties, and meal preparation difficulties are common concerns in many aging societies. Future studies in diverse cultural and dietary settings are needed to examine whether similar eating behavior patterns are associated with sarcopenia in other populations.

## 5 Limitations

Although the present study reveals important findings, it has several limitations. First, the study only investigated the frequency of food group intake using the DVS, without measuring actual food intake. While DVS has been shown to correlate with the quality of the Japanese diet [27] and can reflect dietary content, a detailed quantitative dietary survey is necessary to fully assess nutritional status. Therefore, total energy intake, protein intake, and other nutrient intakes could not be determined. As a result, the nutritional mechanisms underlying the observed associations remain speculative and should be interpreted with caution. Additionally, as this was a cross‐sectional study, the causal relationships between eating behavior patterns and sarcopenia could not be established. Reverse causality is possible; for example, older adults with sarcopenia or reduced physical function may experience appetite loss, eating difficulties, or meal preparation difficulties. Further longitudinal studies are required to explore changes in eating behaviors over time and their impact on future health risks. Second, latent class analysis involves classification uncertainty, and class membership should be interpreted as probabilistic rather than deterministic. However, the four‐class solution was selected based on model fit, class size, and interpretability. Third, participants were community‐dwelling older adults from one Japanese municipality, which may limit generalizability to other settings and populations.

## 6 Conclusion

The eating behavior patterns of older adults are influenced by factors such as meal preparation difficulties, food quantity, and motivation to eat, and these patterns are associated with specific food group intake frequencies and sarcopenia. The findings suggest that categorizing eating behavior patterns may help identify older adults who could benefit from nutritional assessment and support for community‐dwelling older adults. In addition to addressing meal content, providing support for meal preparation and increasing motivation to eat are potentially relevant approaches for nutritional support in older adults with sarcopenia‐related vulnerability. Future longitudinal and intervention studies are needed to clarify causal relationships and evaluate whether tailored nutritional support based on eating behavior patterns can contribute to sarcopenia prevention.

## Author Contributions

**Tomoki Tanaka:** conceptualization, investigation, funding acquisition, writing – original draft, methodology, validation, visualization, software, formal analysis, data curation, writing – review and editing. **Fumika Kamijo:** writing – review and editing, investigation, conceptualization, writing – original draft, methodology, formal analysis, software, data curation. **Hiroko Ito:** conceptualization, investigation, methodology, funding acquisition, writing – review and editing, supervision, resources. **Koji Kurata:** conceptualization, investigation, writing – review and editing, visualization. **Katsuya Iijima:** conceptualization, investigation, funding acquisition, writing – review and editing, visualization, methodology, validation, supervision, formal analysis, project administration.

## Ethics Statement

The study protocol was approved by the ethics committee of the University of Tokyo (#21‐192). The study adhered to the Strengthening the Reporting of Observational Studies in Epidemiology (STROBE) guidelines for cohort studies and was conducted in accordance with the principles of the Declaration of Helsinki.

## Conflicts of Interest

The authors declare no conflicts of interest. F.K., H.I., and K.K. are affiliated with Kewpie Corporation. Kewpie Corporation did not provide funding for this study and had no institutional role in the study design, participant recruitment, data collection, data analysis, interpretation of the findings, or the decision to submit the manuscript for publication. The contributions of authors affiliated with Kewpie Corporation were made in their capacity as coauthors and in accordance with authorship criteria.

## Transparency Statement

Katsuya Iijima affirms that this manuscript is an honest, accurate, and transparent account of the study being reported; that no important aspects of the study have been omitted; and that any discrepancies from the study as planned have been explained.

## Supporting information


Supporting File

## Data Availability

The data that support the findings of this study are available from the corresponding author upon reasonable request.
