# Supplementary material for: Eating Behavior Patterns, Food Group Intake Frequency, and Sarcopenia in Community‐Dwelling Older Adults: A Cross‐Sectional Study From the Kashiwa Study
Source: Health Sci Rep. 2026 Jul 16;9(7):e72832. doi: 10.1002/hsr2.72832 (PMC13376594; doi:10.1002/hsr2.72832)
Supplement: Supplementary file 1 — Supporting File [file HSR2-9-e72832-s001.docx]

**Supplementary information**

Eating Behavior Patterns, Food Group Intake Frequency, and Sarcopenia in Community-Dwelling Older Adults: A Cross-Sectional Study from the Kashiwa Study

**Supplementary Table 1.** Model fit indices for candidate latent class models

| Number of classes | −Log likelihood | Number of parameters | AIC | BIC | Smallest estimated class proportion | Decision^*^ |
| --- | --- | --- | --- | --- | --- | --- |
| 2 | 12692.22 | 37 | 25458.4 | 25665.7 | 17.7% | Not selected |
| 3 | 12592.14 | 56 | 25296.3 | **25610.0** | 3.9% | Not selected |
| 4 | 12527.10 | 75 | 25204.2 | 25624.4 | 3.7% | Selected |
| 5 | 12483.17 | 94 | **25154.3** | 25681.0 | 3.4% | Not selected |

*Notes:* AIC, Akaike information criterion; BIC, Bayesian information criterion.

^*^ The three-class solution showed the lowest BIC, whereas the five-class solution showed the lowest AIC. The four-class solution was selected based on the overall balance of model fit, class size, and clinical interpretability. In particular, the four-class model enabled identification of clinically meaningful eating behavior profiles, including a distinct preparation-difficulty type, without excessive fragmentation of classes.

**Supplementary Table 2.** Conditional response probabilities for the selected four-class latent class model

|  |  | Cluster 1 | Cluster 2 | Cluster 3 | Cluster 4 |
| --- | --- | --- | --- | --- | --- |
|  | n (%) | 1299 (64.8) | 400 (20.0) | 229 (11.4) | 76 (3.8) |
| Daily food intake | A lot | 0.0% | **82.3%** | 0.0% | 13.2% |
|  | Normal | **94.2%** | 17.8% | 14.4% | 67.1% |
|  | A little | 5.8% | 0.0% | **85.6%** | **19.7%** |
| Snack amount | A lot | 3.4% | **34.0%** | 10.0% | 6.6% |
|  | Normal | **52.4%** | 35.3% | 19.2% | **51.3%** |
|  | A little | 44.2% | 30.8% | **70.7%** | 42.1% |
| Eating speed | Fast | 32.4% | **64.8%** | 12.7% | 36.8% |
|  | Normal | **51.2%** | 28.3% | 31.0% | 40.8% |
|  | Slow | 16.4% | 7.0% | **56.3%** | **22.4%** |
| Number of meals per day | 1-2 | 3.1% | 3.8% | **7.0%** | **5.3%** |
|  | 3 | 96.8% | 95.8% | 90.4% | 93.4% |
|  | 4 or more | 0.1% | 0.5% | **2.6%** | **1.3%** |
| Drinking habits | Yes | 49.3% | **51.3%** | 41.9% | 22.4% |
|  | Quit | 4.1% | 5.0% | **6.6%** | **14.5%** |
|  | No | 46.7% | 43.8% | 51.5% | **63.2%** |
| Takes supplements | | 51.4% | 50.8% | **55.9%** | 47.4% |
| Has difficulties shopping |  | 0.8% | 1.8% | 3.5% | **100.0%** |
| Has difficulties getting ready |  | 0.4% | 5.0% | 3.9% | **60.5%** |
| Cannot prepare meals alone | | 3.7% | **4.5%** | **7.0%** | 1.3% |
| Eats alone |  | 10.9% | 18.0% | **30.1%** | 30.3% |
| Eats with friends less than once a month | | 34.4% | 33.3% | **46.3%** | 34.2% |
| No appetite |  | 0.4% | 0.0% | **14.0%** | **4.0%** |
| Does not enjoy meals |  | 0.1% | 1.0% | **15.7%** | **17.1%** |

**Bold numbers：**Items with a response probability of 5% or higher than the total composition rate.
